# Numerical Simulation of Na-Tech Cascading Disasters in a Large Oil Depot

**DOI:** 10.3390/ijerph17228620

**Published:** 2020-11-20

**Authors:** Shaobiao Zhang, Dayong Xu, Gansu Shen, Junguo Liu, Lili Yang

**Affiliations:** 1 School of Environment, Harbin Institute of Technology, Harbin 150090, China; zsb@szsti.org; 2 Department of Statistics and Data Science, Southern University of Science and Technology, Shenzhen 518055, China; 3 Shenzhen Urban Public Safety and Technology Institute, Shenzhen 518046, China; xudy@szsti.org (D.X.); shengs@szsti.org (G.S.); 4 College of Safety Science and Engineering, Nanjing Tech University, Nanjing 211816, China; 5 School of Environmental Science and Engineering, Southern University of Science and Technology, Shenzhen 518055, China

**Keywords:** cascading disaster, numerical simulation, large oil depot, landslide, vapor cloud explosion

## Abstract

The mechanism of natural-hazard-triggered technological (Na-tech) cascading disasters is complex, and the extent to which their damage is aggravated by various secondary events is difficult to quantify. This study selected a large oil depot and constructed a full-scale three-dimensional scene model based on the surrounding geographical environment. The discrete element method (DEM), finite element method (FEM) and finite volume method (FVM) were employed to conduct numerical simulations of the process and consequences of the following Na-tech disasters: heavy-rainfall-induced landslide → blocks impacting an oil transportation pipeline and breaking it → oil leaking, spreading and resulting in a vapor cloud explosion. According to the results, the maximum impact of the 1 m^3^ of sliding mass formed in the landslide on the pipeline was over 7 MN (meganewton), and the pipeline fractured completely when it was loaded with a contact force of only 1.44 MN. The numerical simulation methods revealed the mechanism of Na-tech cascading disasters in a large oil depot and quantified the consequences of each event in the cascading disasters.

## 1. Introduction

China is among the countries suffering most from natural disasters. The way and extent to which natural disasters affect urban security are transforming, resulting in much more complicated causalities caused by cascading disasters. The complexity of cascading disasters, exacerbated by the changing qualitative and quantitative characteristics of these natural disasters, is threatening urban security. One of the sources of the increasing complexity is the rising inherent risk of natural disasters; another is the over-exploitation of natural resources by humans during the process of urbanization. Natural-hazard-triggered technological (Na-tech) cascading disasters have attracted increasing attention of scientific researchers. Landslides, for example, have been intensively studied by scholars who have tried to elucidate their mechanisms through numerical simulations.

Rainfall-triggered landslides are typical cascading disasters which have been studied by many researchers. Cui et al. [1] used three different methods to estimate the peak flow at different locations and analyzed the key modes of cascading landslide dam failure. The results showed that the complex cascading of geomorphic events (landslide dam failure and inclined bed erosion) can be extended to catastrophic emissions. Zhang et al. [2] developed the iCRESTRIGRS model by integrating the coupled routing and overflow coupling (CREST) model with the physics-based TransientRainfall infiltration and grid-based regional slope stability (TRIGRS) landslides and evaluated the iCRESTRIGRS model in the case of Western North Carolina. The overall accuracy of the coupled model was 98.9%, and the true positive rate was 56.4%. Kim et al. [3] studied the instability of shallow landslides on the Umyeonsan mountain caused by rainfall through a coupled hydro-mechanical finite element model. By comparison with field surveys, this method seems more reasonable than limit equilibrium method in determining the landslide launch Potential area. Keijsers et al. [4] optimized the spatial explicit prediction of landslide hazards in Taiwan’s mountains indicating that the prediction of landslide location is more accurate with additional incorporated spatial data. By introducing a critical rainfall threshold, Papa et al. [5] modeled the infiltration of land through a simplified Richards equation, and studied the relationship between the critical rainfall threshold and landslides. In addition, many studies focused on earthquake-type landslides. Jibson et al. [6] employed Newmark method to predict approximate landslide displacement. The seismic acceleration time history was selected to represent the vibration conditions of interest, and those parts of the records higher than the critical acceleration were integrated twice to determine the permanent landslide displacement. Keefer [7] used regression and one-way analysis of variance (ANOVA) techniques to study the landslide caused by the Loma Prieta earthquake in California in 1989. The results showed that the landslide density (defined as the number of landslide sources per unit area) is negatively correlated to its distance to the earthquake source while positively correlated with steepness of the slope There are great differences in landslide density among various geological units in this area. some other researchers [8,9,10,11] also have tried to determine the hazard resulting in models that associate geological and hydrological conditions and allow the evaluation of the temporal probability.

**Figure 1:**
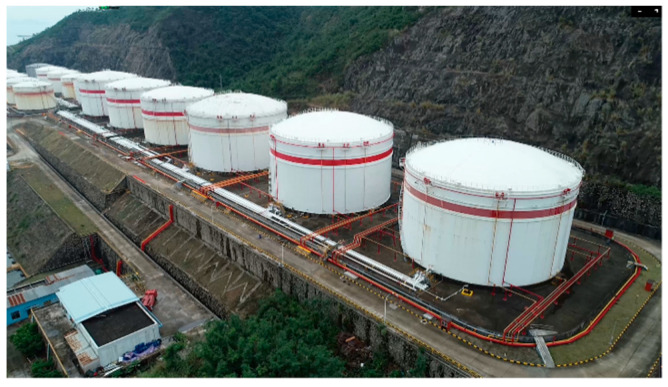
Slope conditions for an oil depot.

Relevant literature is mainly focused on the cause and process of landslides or intensively discusses cascading landslide-induced debris flow disasters with a focus on the influences of debris flows on villages or roads instead of industrial facilities [12,13]. However, in real-world situations, landslides can cause damages to facilities, including oil and gas transportation pipelines, gas stations, and oil and gas storage tanks and such damages can lead to fires and explosions. To our knowledge, no study has conducted any numerical simulation of the entire chain of cascading disasters involving oil and gas transportation pipelines damaged by landslides. Considering the potential risk of disasters in medium and large sized oil and gas storage areas in cities, we designed a cascading disaster scenario according to the actual situation of a large oil depot, the full-scale modeled environment, and the actual terrain conditions (Figure 1). In the scenario, landslide result in structural damage and leakage of a gas pipeline, which subsequently leads to the formation of flammable vapor clouds and then an explosion.

## 2. Scene Construction

**Figure 2:**
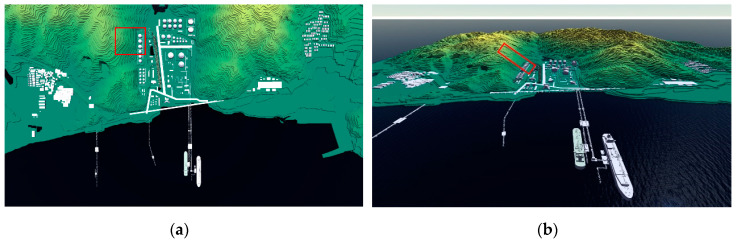
3D scene model of the study site: (**a**) Top view; (**b**) Side view.

Scene construction is a process of integrating the actual features (including buildings, structures, facilities, vegetation, rivers, oceans, and geographical features) of environment into the numerical simulation of a disaster-affected object. The three-dimensional (3D) scene constructed in this study encompasses the following features: 9 km^2^ digital elevation model, large oil depot, pipelines, oil transportation station, office, two communities located on the east and west sides of the mountain, roads, trees, harbor, rivers, ships, and coastal waters (Figure 2). The area marked with a red box is the sliding block.

In this paper, the DEM (discrete element method) approach was employed using 3 Dimension Distinct Element Code (3DEC), with consideration of the complex hydromechanical coupling effects, to simulate three block size conditions to determine the influence of block size on the speed of sliding block movement and level of impact. The output data, namely the contact force between the block and pipeline, effective contact time, and area of contact were used as the initial loading conditions in the finite element analysis of nonstructural damage to the pipeline by LS-DYNA. Subsequently, we calculated the deformation process of fractures in the pipeline impacted by blocks rolling down the slope. Finally, the pipeline deformation result was adopted as the basis for calculating the amount of leakage from the pipeline, which was used to simulate the formation of flammable vapor clouds and explosions by flame acceleration simulator (FLACS). As one of the best commercial software in their respective fields, 3DEC [14,15], FLACS [16], and LS-DYNA [17] have all been verified by a large amount of experimental data.

## 3. Analysis of Heavy-Rainfall-Induced Slope Deformation and Damage

The research site is located on the east side of Wutong Mountain (Shenzhen, China). The average annual rainfall in the region is 1933.2 mm, the maximum annual rainfall is 2662.2 mm (1975), and the minimum annual rainfall is 912.5 mm (1963). The rainy season lasts from May to September, and it accounts for 78% of the annual rainfall. From October to April is the dry season, accounting for 22% of the annual rainfall. The number of average annual rainy day is 144.7 with a maximum daily rainfall of 203 mm (27 June 2001). The rock-soil body of the landslide is composed of sand gravel, highly weathered coarse biotite granite, and slightly weathered coarse biotite granite. During heavy rainfall, the water level in the aquitard rises continuously due to its inability to discharge rainwater in a timely fashion, which considerably increases the weight and sliding force of the sliding blocks. Each block’s strength and loading determine the corresponding decrease of target strength as the effective stress decreases. Additionally, rainfall may result in the water wedge effect, enlarging cracks in the block [18]. Accordingly, the mass becomes unstable and approaches a state of failure. As the mass moves, its collision and friction losses are reduced by rainwater, which maintains a high level of kinetic energy of the mass and thus increases its impact on engineering structures.

It should be noted that in the analysis, the rainfall infiltration process was not directly simulated because of the complex mechanical mechanism of rain-induced landslide [19], instead under the initial equilibrium state of the model, the residual strength parameters of the contacts between blocks were evaluated, which forces the landslide to change from the natural stable state into the motion failure. This can be regarded as an equivalent effect of the trigger of the rain-induced landslides. Affected by the internal structure and terrain control, most landslides disintegrate into blocks once they become unstable, and the blocks move randomly with variable speeds and trajectories [20]. The friction and collision between blocks during the movement causes energy consumption and conversion, ultimately causing the blocks to stop, and the distance of their movement determines a landslide’s magnitude of influence. According to research, blocks in mountain landslides move at higher speeds and impact production facilities at the bottom of the mountain, causing catastrophic damages.

### 3.1. Method

The most commonly used methods for simulating landslides include DEM, FEM, FVM, and smoothed-particle hydrodynamics (SPH). In particularly, FVM and SPH originate from the field of hydrodynamics and have been increasingly employed to solve slope stability problems. Geographical phenomena (including landslides) generally exhibit intrinsic mechanical properties such as geometric discontinuity, material inhomogeneity, and nonlinearity. FEM, FVM, and SPH are all based on continuum mechanics and cannot be used to simulate the complex discontinuous behavior after slope instability, hence inapplicable to such phenomena. 

Compared with continuum mechanics methods, DEM features the ability to describe the continuum as well as discontinuous mechanical behavior of contact and thus to effectively process discontinuous geographical surface [21,22]. The DEM analysis of rocks, for example, involves categorizing rocks into two basic subjects: rock mass (continuum) and structural plane (contact), with structural plane being the boundary of the rock mass. This way, each continuum is treated as an independent subject in the mechanical calculation to obtain its mechanical response according to the continuum theory; the mechanical relationship between each continuum is determined according to the nonmechanical behavior of the boundary (contact).

### 3.2. Model Parameter Setting

In 3DEC, the rock mass is simplified as a set of polyhedron blocks connected by specific contact models and the block could be deformable through meshing into finite-difference tetrahedral elements internally. The proposed numerical model incorporated geographical data including bedrock slope and potential sliding mass. Bedrock slope was considered collectively as a superblock Wall rigid block made of tetrahedrons, and the sliding mass, as the target of analysis, was converted into an aggregate of deformable tetrahedron block. The interaction effect between blocks in the model was described using a contact constitutive model based on Mohr-Coulomb failure [23].

The block density was set at 2700 kg/m^3^. The contact mechanical parameters were directly taken from an existing research of another landslide in the same area [24], the Guanghui oil depot. The initial and residual strengths quantified the natural strength and the strength at which failure occurs, respectively. According to the internal structure of a deposit, the initial strength reflects the natural mechanical properties of cement, which enhances the loading strength of a sliding mass and thus maintains its natural stability. When rainfall causes sliding and failure of a mass, the cementation between blocks is destroyed completely by the friction between blocks and rain erosion [25]. Subsequently, interaction (including collision) occurs between the blocks through direct contact on their surface, and thus the strength conditions were determined by the strength of the surface of rock mass. Considering the surface properties of rock blocks and rainwater’s infiltration and lubrication in the rock blocks and ground surface, we calculated a conservative determination of the residual (ultimate) strength.

This study’s discussion is based on the assumptions that the sliding mass is a deposit formed on a bedrock surface for certain geographical reasons and that the cracks in blocks are filled with cement. The sliding mass was discretized into an aggregate of tetrahedral blocks with a given size (side length), and the filler was described using the contact between blocks. Because a sliding mass was formed with tetrahedral blocks of randomly varying sizes, a stable sliding mass approximated to an isotropic quasi-continuum. We assumed the tetrahedral blocks to be rigid bodies and that the blocks do not fracture or disintegrate during collision with one another. 

The model incorporated a comprehensive range of engineering structures, such as oil storage tanks, primary and secondary pipelines, security fencing, and retaining wall, in the engineering area below the slope where the studied sliding mass was located [26]. Considering that structural analysis is not the strength of the 3DEC, we treated these structures as rigid bodies in the analysis and simulated the impact of sliding blocks on engineering structures according to the variable contact between them.

The general rule of discrete element model construction is that it must maintain an effective balance between calculation efficiency and accuracy. The core content to be solved in the study’s DEM iteration was the movement of blocks, contact type between blocks, and loading of blocks; therefore, the size of blocks had a significant influence on the analysis efficiency. A 3DEC process employed an explicit finite-difference scheme and iterative calculation to solve landslide movement problems concerning the real-time (physical time) process of geographical failure. The physical time (critical time step) presented in each iteration was related directly to the physical and mechanical properties of the medium as well as the discrete size.

On the basis of the fundamental assumptions, influence factors of model analysis efficiency, and features of the analysis, we used the following approach to construct models for all targets of analysis except for sliding mass to achieve a balance between efficiency and accuracy. Specifically, because these targets were considered to be rigid bodies and to not involve any movement (i.e., their positions were fixed) in the analysis, each of the targets (e.g., bedrock slope) could be simulated using a superblock WALL. A superblock WALL comprised one or multiple blocks; the contact between blocks within the same WALL or in different WALLs was not determined in the iteration, which greatly reduced the amount of model calculation and thus enhanced the analysis efficiency.

**Figure 3:**
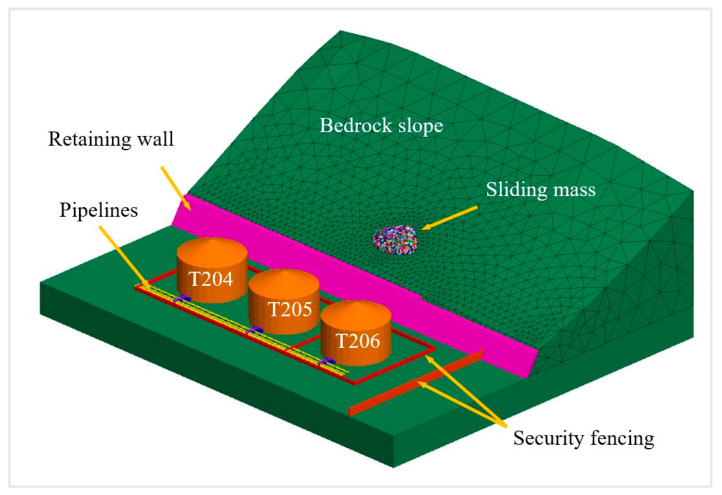
Model of slope and engineering structures.

According to the aforementioned principles, a model of the studied slope and engineering structures was constructed using a 3D geographical model and block discretization (Figure 3). The deposit (sliding mass) was located on the bedrock slope at the elevation of 35–50 m and had a total volume of approximately 1000 m^3^.

Studies on landslide movement have predominantly investigated the influences of the structural characteristics and strength conditions of a sliding mass on its movement. For example, Yoichi Okura et al. [27] placed multiple stones on a slope to examine the influence of stone size on the stones’ sliding distance along the slope and verified said influence of stone size.

The DEM has an edge over other analysis methods in describing authentically the internal structure conditions of a sliding mass. Accordingly, we established three Cases with different block sizes according to the sliding mass model in Figure 3 to investigate the influence of block size conditions on the movement of sliding mass and its impact on the engineering structures. Table 1 illustrates the parameter setting according to the reference [27,28]; Case A had the smallest block size in the sliding mass (mean block size = 0.54 m^3^), followed by Case B, and then Case C.

We defined mechanical parameters for the sliding mass in the model according to Table 2 [26,29] and concurrently incorporated self-load into the model. After necessary boundary conditions had been determined, iterative calculation was conducted until a balance was reached. The contact strength between blocks in the sliding mass was adjusted to be the ultimate strength to induce failure in the sliding mass. Then, a kinetic analysis was conducted to study the process of disintegrative failure. The total calculation time was 30 s, and the damping coefficient was 0.2.

### 3.3. Analysis of the Simulation Result

In Case A, the blocks exhibited diverse movement types, including the four typical types: falling, bouncing, rolling, and sliding. Because the top surface of the retaining wall was nearly in parallel to the ground, which resulted in a sudden change of grade relative to that of the bedrock, some of the blocks moved as projectiles after they dashed against the wall. Some of the blocks, colliding forcefully against the wall, lost their kinetic energy due to energy conversion and thus accumulated at the bottom of the slope.

The storage tanks, whose layout axis was nearly perpendicular to the direction of the sliding mass movement, were easily hit and hence damaged by the blocks. Accordingly, the blocks were also found to accumulate on the ground between the storage tanks and the slope. Because the structure of the storage tanks provided sufficient resistance to the impact, they were beneficial for the protection of the pipelines. For example, the root valve was protected by the storage tanks from being impacted by the blocks.

**Figure 4:**
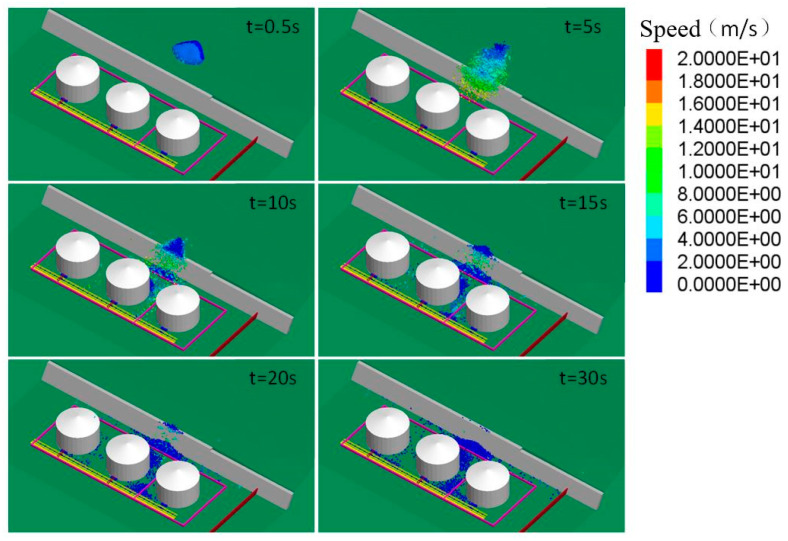
Distribution of blocks at key time points (Case A).

Blocks with a relatively high level of kinetic energy passed through the space between storage tanks and continued moving toward the pipelines. After 10–12 s from when the landslide occurred, the blocks approached, impacted and damaged the pipelines (Figure 4).

**Figure 5:**
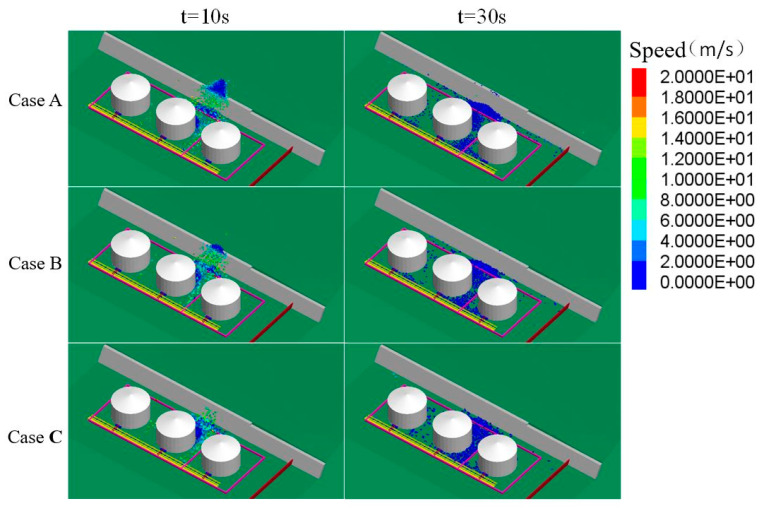
Influence of block size on block movement speed.

Figure 5 compares the block distribution of the three Cases at different time points. When t = 10 s, the surface of the bedrock was the movement boundary for a large amount of blocks in Case A; in Case B, most of the blocks finished their projectile movement initiated by hitting the retaining wall and started to accumulate at the bottom of the slope; in Case C, a larger amount of blocks had accumulated at the same spot.

These results verified the influence of block size on the average speed of block movement. Overall, the movement speed of blocks was directly proportional to the block size. In each of the three Cases, the peak movement speed of blocks was approximately 30–40 m/s, which revealed a high level of kinetic energy.

As we studied the damage caused by blocks, we also monitored the contact between the blocks and pipelines. The contact force between the two could be obtained once the they came in contact. The maximum contact force for each pipeline at each second was extracted and compared with the real physical time to establish an impact curve across time, which facilitated an intuitive understanding of the characteristics of the blocks’ impact on the pipelines.

**Figure 6:**
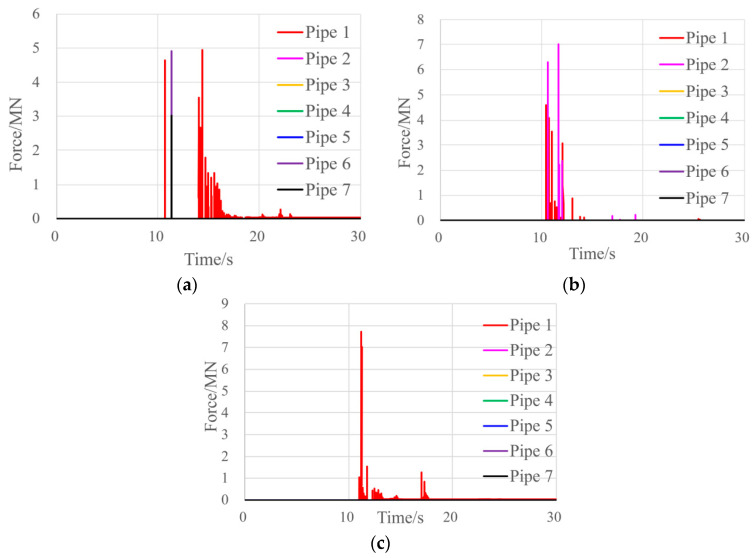
Impact of blocks on pipelines across time: (**a**) Case A; (**b**) Case B; (**c**) Case C.

Figure 6 presents the time-history curve of block impact on pipelines in three Cases, which revealed that the blocks impacted the pipelines at 10–12 s after the landslide occurred in all three conditions. As with the speed of block movement, the level of impact also showed a positive correlation with block size. In Case A, the maximum impact (4.94 MN) was observed in Pipeline 1 (the one located nearest to the slope); the maximum impacts in Cases B and C were 7.0 MN and 7.7 MN, respectively. Because of the uncertain internal structure of the blocks, the three conditions yielded different impact points.

**Figure 7:**
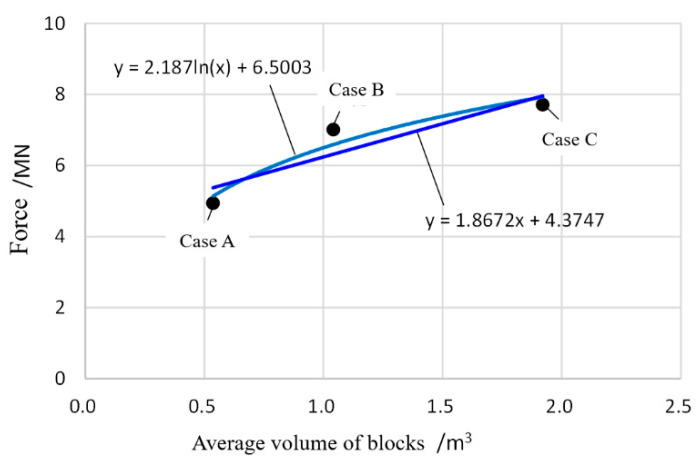
Relationship between maximum impact and block size.

The relationship between the maximum impact and block size was established (Figure 7), and the fitting function was obtained: 

Linear fitting: y = 1.8672x + 4.3747

Logarithmic fitting: y = 2.187ln(x) + 6.5003

## 4. Analysis of Damages in the Oil Transportation Pipeline Structure

When impacted by blocks, gas pipelines can undergo plastic deformation or even fracture in severe cases. Therefore, LS-DYNA was used to reconstruct the structural model of the impacted pipeline sections [22,26]. The block impact load was added to the pipeline structure for accurate calculation of the instantaneous mechanical response when the pipelines received impact, prediction of the pipeline deformation, and evaluation of the existing pipelines’ resistance to impact.

### 4.1. Boundary and Load Conditions Setting

The area with the highest risk was modeled. Pipeline 1 had a diameter of 355 mm, and pipelines 2 and 3 both had 200 mm diameters; the wall thickness was 7.1 mm for all three pipelines. The concrete supports below touched the pipelines at their ground-facing surfaces; the supports were 1322.5 mm high, 350 mm wide, and situated 5000 mm away from each other. The pipelines were made of X52 seamless steel and had yield stress of 360 MPa. We set the time to be 230 ms to investigate the stress state and deformation of the pipelines.

**Figure 8:**
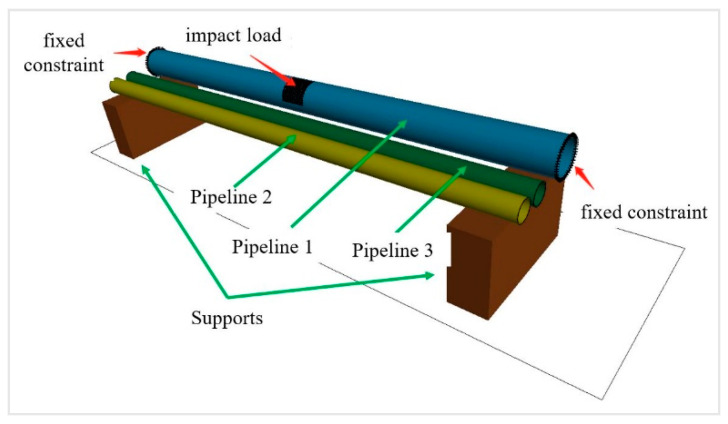
Boundary condition and load.

**Figure 9:**
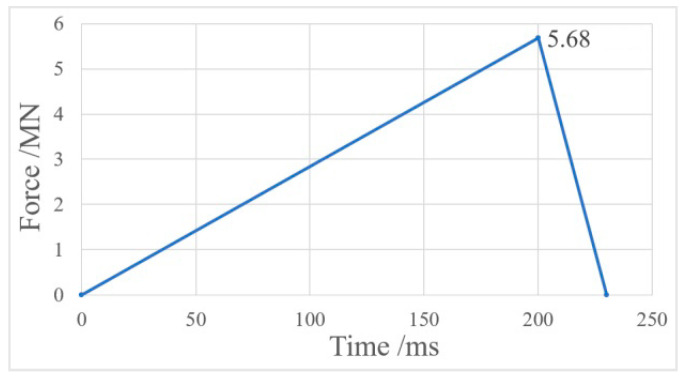
Time-history curve of impact.

Considering that the constructed model included only the feature section of the pipelines, we examined the mechanical response of Pipeline 1 by using fixed constraints at the two ends of Pipeline 1 and applying the impact load on a local area between the two ends. The supports were fixed to the ground and touched the pipelines, and any deformation of the supports was ignored in the analysis (Figure 8). The pipelines fixed on both sides increased the strength of the support, and the pipeline structure is more prone to damage, which is biased towards safety and increases the safety margin. The pipeline adopted the follow-up strengthening material model, the J2 flow plastic theory and equivalent plastic strain failure criterion, which will be able to simulate large plastic deformation and failure behavior of pipe materials [30]. A time-history curve of impact was produced by simulating block impact on pipelines in Case B (Figure 9). The impact lasted 230 ms; the peak impact was 7.1 MN; and the load loss coefficient was determined to be 0.8.

### 4.2. Analysis of the Simulation Results

**Figure 10:**
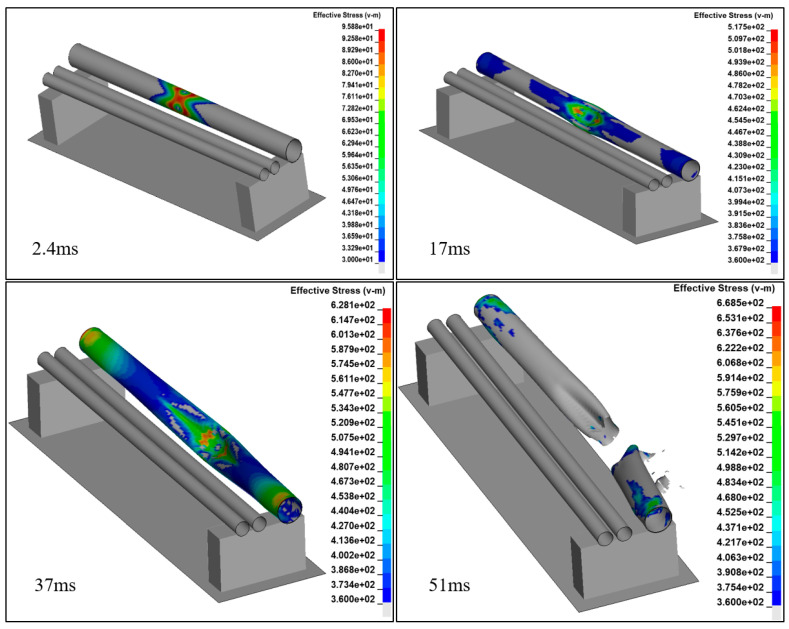
Equivalent stress.

**Figure 11:**
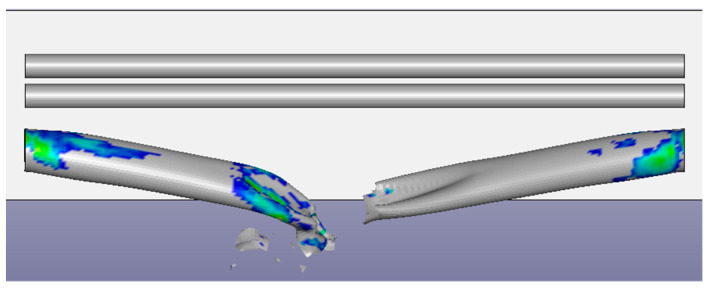
Deformation of the pipeline (51 ms).

Figure 10 shows the finite element analysis result of the impacted pipeline. When the load contact force was at 2.4 ms, the stress was the highest at 95.88 MPa, which was lower than the yield stress of the material. At 17 ms, Pipeline 1 exhibited a large dent, severe plastic deformation, and excessive local stress under the impact, which easily damaged the material and considerably reduced its oil transportation capacity. At 37 ms, most parts of Pipeline 1 reached the state of plasticity, the impacted areas were squashed, and the entire feature section was bent. At this point, this section of Pipeline 1 could barely transport oil and gas, and the flow speed in the squashed area dropped, leading to a pressure surge in this area. At 51 ms, the excessively large impact damaged the material at the impacted positions, the material lost its load capacity, and ultimately the pipeline fractured; oil and gas leaked out from the fracture points (Figure 11).

According to the impact curve, the contact force was only 1.45 MN at 51 ms; specifically, the pipeline fractured after 51 ms from the first impact by blocks, following which the contact force decreased to 0. According to the critical contact force obtained using the FEM, the actual loss coefficient was 0.204.

## 5. Analysis of Pipeline Leakage Resulting in Flammable Vapor Cloud Explosion

### 5.1. Boundary Conditions Setting

We employed Pool Model 3 in the FLACS software to simulate the process in which the leaked gasoline gathered into a pool, and the pool expanded as some of the gasoline evaporated. The fluid was set as compressible fluid, and a wall function was used to calculate the near-wall flow field; the substance was determined to be gas with a composition of 9.6% butane, 17.2% pentane, 16% hexane, and 57.2% decane [31]. A large volume of flammable vapor cloud existed when stable atmospheric convection, high ambient temperature, and low wind speed were present. To ensure reliable environmental parameters, we referred to weather data published by the location weather station on its website and determined the environmental parameters to be as follows: temperature = 35.3 °C, wind speed = 1 m/s, wind direction = northwesterly, atmospheric stability = F, solar irradiance = 739 W/m^2^, initial turbulence strength = 0.1, and turbulence length scale = 0.01. The ground roughness was set at 0.01 m according to the geographical characteristics.

**Figure 12:**
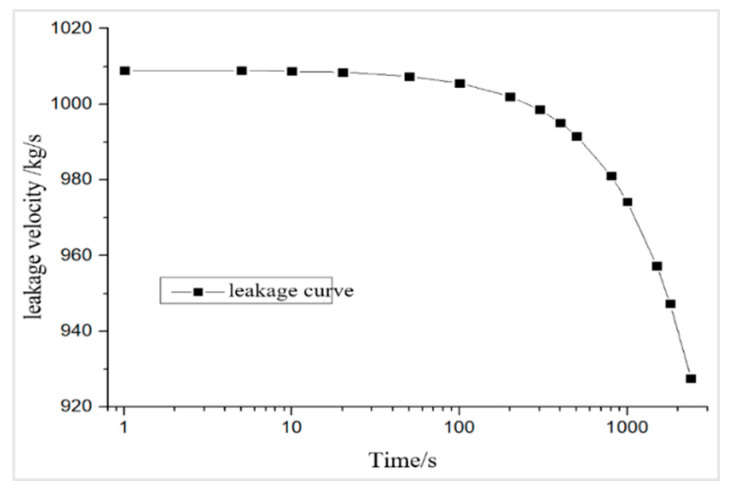
Gas leakage curve.

The boundary condition was WIND on the northwest and NOZZEL for the other directions. According to a damage analysis of the pipeline structure, the pipeline, which was connected to the storage tank, fractured completely. Assuming that the emergency shutdown device (ESD) malfunctioned, we analyzed the instantaneous amount of leakage from the pipeline. The opening from which the gas leaked had an internal diameter of 355 mm, and the center of the opening was 1500 mm above the ground. The storage tank had a design capacity of 20,000 m^3^ and an internal diameter of 37 m, and the density of the gas was determined to be 0.78 g/cm^3^. Equation (1) was employed to calculate the instantaneous leakage:

(1) $$Q_{m}=\rho \bar{u}A=\rho AC_{0}\sqrt{2\left(\frac{g_{c}P_{g}}{\rho }+gh_{L}\right)}$$

where $Q_{m}$ is the instantaneous mass flow rate; ρ is the gas density; $\bar{u}$ is the average instantaneous flow velocity of fluid discharged from the opening; A is the area of the opening; CO is the discharge coefficient, which is set at 1.0 to maximize the flow; $g_{c}$ is the gravitational constant; g is the gravitational acceleration; $h_{L}$ is the vertical distance between the leakage opening and the gas level in the tank; and $P_{g}$ is the gauge pressure above the gas level. When the tank was fully loaded, the relationship between the instantaneous leakage velocity at the opening $Q_{m}$ and the time of leakage t is as presented in Equation (2), which can be used to calculate the leakage velocity at different time points across 40 min to produce a leakage curve (Figure 12):

(2) $$\frac{Q_{m}{}^{2}}{0.05958481}+\frac{Q_{m}\cdot t}{838.2387}=17.11$$

The study site was divided into inner core, outer core, and noncore regions. Table 3 illustrates of the grid size; X and Y are the planar directions, and Z is the elevation. A coefficient of 1.2 was employed between the regions to facilitate a smooth transition, and a total of 13,849,792 grid squares were used.

For the simulation of explosion, the ignition source was set in the fire pumping station and referred to the Buncefield oil depot explosion in the U.K. Assuming that the start-up of the fire pumping station ignited the flammable vapor cloud [32]. The explosion occurred at 2595 s after the leakage began. Given the characteristics of the terrain, surrounding vegetation (trees), and equipment distribution, we considered the possibility of deflagration to detonation transition (DDT) and thus selected the gas explosion model in FLACS to simulate the explosion caused by flammable vapor cloud. This model involved the use of a spatial pressure gradient across the flame front (DPDX) to effectively predict the possibility of DDT. DPDX parameters can be used to capture the timing of peak pressure in the flame front and thus determine whether DDT exists. To ensure that, in each time step, pressure can be transmitted to five control bodies, and fluid can be transmitted to 0.5 control bodies for better results, we set the sound-speed scale (CFLC) and flow-speed scale (CFLV) at 5 and 0.5, respectively. The initial environmental parameters for explosion were consistent with those for the simulation of gas leakage.

The boundary condition was set as PLANE WAVE to effectively simulate the far-field transmission of overpressure blast-wave. On the basis of the grid established for the pool expansion simulation, we increased the grid density to (0.5 × 0.5 × 0.5) m within the 50 m radius of the fire pumping station, with a total of 14,265,422 grid squares constructed.

### 5.2. Simulation of Gasoline Leakage

**Figure 13:**
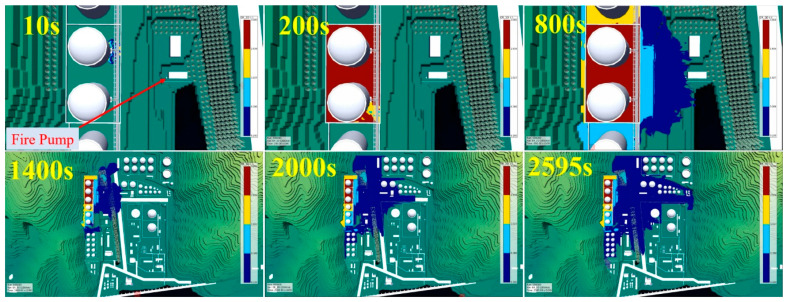
Process of gasoline leakage. (the photographs below contain a larger area than the upper).

According to the simulation result (Figure 13), the gasoline leaked out and gathered into a pool on the ground below the pipeline fracture point. The pool initially concentrated in the northeast part of the region within the firewalls, expanded around the two tanks within the same tank area enclosed by dividing walls, and finally covered the entire region bounded by the firewalls. A total of 315 s elapsed before the leakage covered the entire region bounded by the firewalls, with coverage of 3910 m^2^. The expansion velocity of the vapor was much faster than that of the pool; most of the vapor, due to shielding by the firewalls (1.8 m high), stayed within the firewalls, and the flammable vapor cloud had a volume of only 1383.5 m^3^. Most areas within the firewalls contained vapor concentrations higher than the upper limit of explosion; the amount of gasoline leakage was 315.889 t and that of evaporated gasoline was 9.288 t during this time, reaching an evaporation rate of 2.94%. For verification, the results were compared with the reference [33], which suggested that, when the temperature was 30 °C and the wind speed was 1.0 m/s, gasoline evaporated at a speed of 8 g/(s·m^2^); giving a gasoline leakage expansion of 3910 m^2^ (315 s), this translates into a total evaporation volume of 9.853 t, which is very close to the simulation result, 9.288 t.

If the leakage continues and goes beyond control, and the gas pool grows deeper, the high-concentration gas vapor will surely spread out beyond the firewalls. Because the northern dividing wall (1.5 m high) for the tank area where the leaking pipeline was located was shorter than the firewall, the high-concentration vapor first crossed the dividing wall and concentrated in the tank area to the north of the tank. Additionally, a high concentration of vapor was observed in the space between the slope and the cofferdam situated west to the leaking tank. At 800 s, the high-concentration vapor cloud floated toward the fire station and pumping station located at a lower elevation, revealing itself to be a heavy gas vapor cloud as it expanded. At 1400 s, the vapor cloud expanded to the greenbelt on the east, filling station on the northeast, and 5000 m^3^ gas tank area on the south. The leakage lasted for 2595 s and resulted in a total gas leakage of 2320.6 t, oil and gas evaporation of 42.8 t (evaporation rate = 1.84%), and flammable vapor cloud of 31,304 m^3^.

### 5.3. Simulation of Explosion

**Figure 14:**
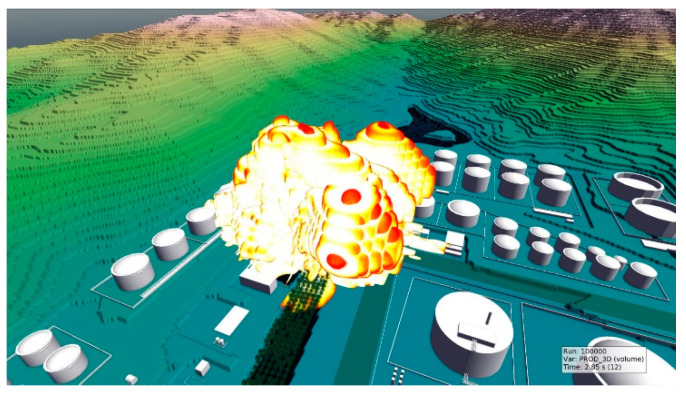
Explosion at t = 2.85 s.

The explosion lasted 2.85 s. Products of the flammable-vapor-cloud-induced explosion were used to reveal the process of explosion (Figure 14). The explosion reached up to 150 m altitude in the explosion site and affected storage tanks in all directions and buildings on the south.

According to the Guidelines for Quantitative Risk Assessment of Chemical Enterprises [34], we categorized the influence of overpressure on humans, buildings, and structures (equipment) as shown in Table 4 and Table 5.

**Figure 15:**
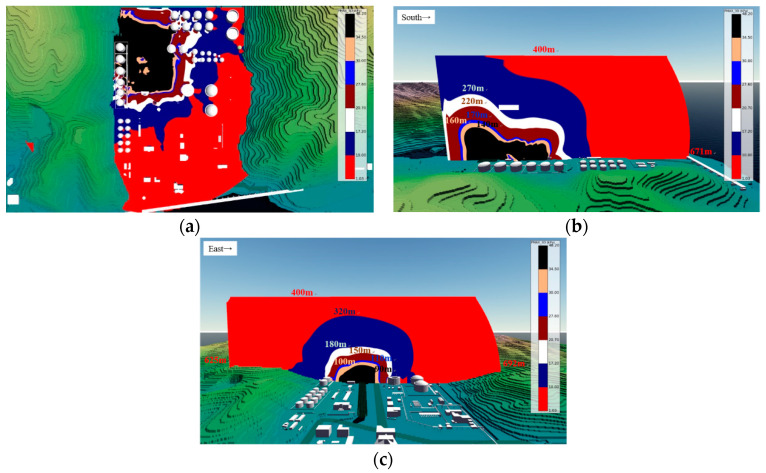
Cross-section of overpressure distribution: (**a**) 19-m-altitude horizontal cross-section; (**b**) North–south cross-section; (**c**) East–west cross-section.

The overpressure generated during flammable vapor cloud explosion is the primary cause of damage. In an open space, overpressure decreases gradually when it is transmitted toward the far field; specifically, when no barriers are present, a location farther from the explosion site exhibits smaller overpressure. According to Table 4 and Table 5, we classified overpressure according to the threshold values; seven overpressure intervals were determined and represented in different colors. For an intuitive presentation of the overpressure distribution, we generated a 19-m-altitude horizontal cross-section of the overpressure distribution from a (Figure 15a). An overpressure of more than 1.03 kPa was transmitted to an altitude higher than 400 m (beyond the study site). Figure 15b is the north–south cross-section passing through the center of the fire pumping station; the overpressure was transmitted to the southernmost point at 671 m. Figure 15c presents the east–west cross-section passing through the center of the LNG storage tank and shows that the farthest points the overpressure was transmitted to were 692 m to the east and 625 m to the west.

**Figure 16:**
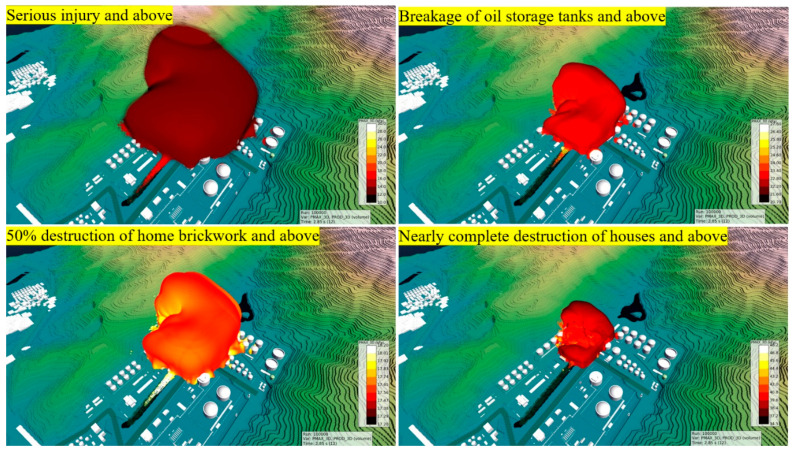
Area of damage by explosion overpressure.

According to the standards for overpressure damage, we output the damaged areas corresponding to their level of damage (Figure 16). Personnel in a large area sustained serious injury or death; relative to the fire pumping station, that area extended 250 m to the north and 150 m to the south, east, and west. Fourteen storage tanks near the pumping station broke or experienced more severe damage, and the five buildings located on the south, north, and east of the pumping station were nearly completely destroyed. Accordingly, in cascading disasters, the secondary events typically lead to more severe damage than do the primary events.

## 6. Conclusions

This study employed heavy-rainfall-induced slope deformation and failure as the initial conditions for cascading disasters and used DEM, FEM, and FVM to conduct a numerical simulation of how an initial disaster triggers a series of disasters. This study demonstrated a research method for studying the mechanism of Na-tech cascading disasters in a large oil depot. Main concluding include as follows:

(1) The DEM method was used to simulate the movement of blocks in a rain-induced landslide and to produce a time-history curve of contact force when the blocks impacted the oil pipeline. The blocks that disintegrated from a sliding mass had a maximum rolling speed of 30 m/s and broke the pipeline at the bottom of the slope in 10–12 s from when the landslide occurred, with the largest contact force being over 7 MN.
(2) The FEM was employed to simulate the nonlinear failure of the pipeline when it was hit by the blocks. According to the contact force curve, we incorporated impact load into the simulation and observed that the pipeline fractured completely at 51 ms after impacted; the actual critical load was 1.45 MN, which was only 20% of the maximum contact force during impacted by the blocks.
(3) The FVM was used to simulate the process in which the gasoline leaked out from where the pipeline fractured and gathered into a pool; the pool expanded while the gas evaporated; the evaporated gas formed a flammable vapor cloud; and the cloud caused an explosion. According to the simulation results, the gasoline leaked out from the fracture point; the gasoline covered the entire area enclosed by the firewalls where the fractured pipeline was located after 200 s of leakage and then spread to other areas. After 2595 s of leakage, the gasoline spread nearly 400 m to the south, more than 450 m to the east, and more than 200 m to the north, reaching the retaining wall for the northern slope.
(4) An ignition source was simulated in the fire pumping station located at the right of the leaking pipeline, and this ignition source resulted in an explosion with a blast radius of more than 150 m. According to the overpressure damage standards and the distribution of personnel, equipment, and buildings on-site, we determined the magnitude of damage by the explosion overpressure to personnel, equipment and buildings.
(5) For large oil depots with side slopes around, geometrical information of the slopes should be obtained through LIDAR scanning. Based on the analysis of slope deformation by LIDAR, dynamic risk assessment is to be conducted. Reinforcement measures should be taken for slopes with higher risk level, and sensors should be installed to facilitate risk-monitoring and warning issues, especially inspections coped with rainstorms. Additionally, scenarios of oil and gas leakage accidents should be constructed according to the reference [36], so as to identify risk factors that may cause derivative disasters. Pump houses, for instance, may constitute ignition sources, hence corresponding explosion-proof measures should be taken.

## Figures and Tables

**Table 1:** Analysis of the Cases.

Block Size Indicator	Case A	Case B	Case C
Maximum size/m^3^	1.42	2.40	3.70
Minimum size/m^3^	0.18	0.38	0.37
Mean size/m^3^	0.54	1.04	1.92

**Table 2:** Parameters for contact mechanics.

1. Rigidity Conditions					
Normal Stiffness			Shear Stiffness		
kn/GPa			ks/GPa		
5.0			2.5		
**2. Strength Conditions**					
**Initial Strength**			**Residual Strength**		
Adhesion C/MPa	Friction angle/o	Tensile strength σt/Mpa	Adhesion C/MPa	Friction angle/o	Tensile strength σt/MPa
0.1	30	0	0	15	0

**Table 3:** Grid size of leakage simulation.

Region	Scale of Region	Grid Size/m		
		X	Y	Z
Inner core	XY: within the firewalls where the leaking pipeline was located (108 × 60 m)Z: 25 m above the ground	0.5	0.5	0.3
Outer core	XY: within the radius of 150 m Z: 25–70 m	1	1	1
Noncore	Other regions	5	5	5

**Table 4:** Damage of overpressure to human.

Overpressure/kPa	Area
2–10	Area of minor injury
10–30	Area of serious injury
≥30	Area of death

**Table 5:** Influence of overpressure on buildings and structures (equipment).

Overpressure/kPa	Damage Description
1.03	Typical pressure for glass breakage
17.2	50% destruction of home brickwork
20.7–27.6	Breakage of oil storage tanks
34.5–48.2	Nearly complete destruction of houses
30,000 [35]	Breakage of Liquified natural gas (LNG) storage tanks
